# Current and Emerging Approaches for Spine Tumor Treatment

**DOI:** 10.3390/ijms232415680

**Published:** 2022-12-10

**Authors:** Bogdan Costăchescu, Adelina-Gabriela Niculescu, Bogdan Florin Iliescu, Marius Gabriel Dabija, Alexandru Mihai Grumezescu, Daniel Rotariu

**Affiliations:** 1 “Gr. T. Popa” University of Medicine and Pharmacy, 700115 Iasi, Romania; 2 “Prof. Dr. N. Oblu” Emergency Clinical Hospital, 700309 Iasi, Romania; 3 Research Institute of the University of Bucharest—ICUB, University of Bucharest, 050657 Bucharest, Romania; 4 Department of Science and Engineering of Oxide Materials and Nanomaterials, Politehnica University of Bucharest, 011061 Bucharest, Romania; 5 Academy of Romanian Scientists, Ilfov No. 3, 050044 Bucharest, Romania

**Keywords:** primary spinal tumors, spinal metastases, spine tumor treatment, interdisciplinary therapeutic approaches, tumor-targeted therapies, custom-made implants

## Abstract

Spine tumors represent a significant social and medical problem, affecting the quality of life of thousands of patients and imposing a burden on healthcare systems worldwide. Encompassing a wide range of diseases, spine tumors require prompt multidisciplinary treatment strategies, being mainly approached through chemotherapy, radiotherapy, and surgical interventions, either alone or in various combinations. However, these conventional tactics exhibit a series of drawbacks (e.g., multidrug resistance, tumor recurrence, systemic adverse effects, invasiveness, formation of large bone defects) which limit their application and efficacy. Therefore, recent research focused on finding better treatment alternatives by utilizing modern technologies to overcome the challenges associated with conventional treatments. In this context, the present paper aims to describe the types of spine tumors and the most common current treatment alternatives, further detailing the recent developments in anticancer nanoformulations, personalized implants, and enhanced surgical techniques.

## 1. Introduction

The spine represents the bony structure housing the spinal cord, which, in addition to protecting this essential part of the central nervous system, is responsible for supporting body weight, withstanding external forces, and allowing for mobility and flexibility while dissipating energy and protecting against impact [1,2]. Unfortunately, the spine is also prone to various diseases, spinal disorders being among the most frequent and expensive medical conditions [3]. The spine in particular was noted to be the most common site of metastases within the skeletal system and a rare but challenging region for several primary tumors that may result in neurological deficits, posing an important burden on patients and healthcare systems worldwide [4,5].

A broad spectrum of treatments is available for spine tumors, ranging from radiation to highly invasive en bloc resection [6]. However, spine oncology therapeutic strategies exhibit certain drawbacks that limit their application and efficacy. Therefore, modern technologies such as nanotechnology, 3D printing, and digital tools started being increasingly used in spine tumor management to overcome the disadvantages associated with conventional treatment approaches.

Most chemotherapeutic drugs encounter obstacles in reaching the target tissue and exerting their pharmacological activity due to the blood–spinal cord barrier, instability of antitumor agents, and rapid elimination from the desired tissue. Moreover, the lack of selectivity renders the organism vulnerable to off-target toxicity [7,8]. In this context, nanomaterials emerged as promising carriers for various drugs, reducing side effects, enhancing drug distribution and bioavailability, improving absorption at the tumor site, and increasing the therapeutic efficacy of transported biomolecules [9,10,11,12,13,14].

Several surgical techniques can be employed when tumor excision is required, depending on tumor type, surgery goal, and overall patient health status [6,15]. To improve procedure precision and postoperative outcomes, emerging digital technologies started being incorporated into tumor resections as complementary instruments for visualizing the surgical field [16]. Moreover, important advances have been made in reconstructive strategies following tumor excision. Recent focus has been oriented toward addressing the specific characteristics of each patient and designing unique personalized implants with the aid of 3D printing technologies [17,18,19].

In this respect, this paper aims to present the newest available information on spine tumors, detailing their types and the most common current treatment options, further focusing on the recent advancements in anticancer nanoformulations, personalized implants, and enhanced surgical techniques. Some of these topics have been the subject of previous reviews in the field [20,21,22,23,24]. However, this paper focuses on the most recent developments in spine tumor treatment, mostly discussing studies published between 2018 and 2022 and indicating several future perspectives. Through its comprehensive approach, this review aims to overview current and emerging therapeutic strategies, serving as an inception point for further research in the field and helping to envisage more efficient solutions.

## 2. Spine Tumors

Tumors localized in the spine account for about 15% of all central nervous system tumors. A spinal cord tumor is considered a mass growing within the spinal canal or within the spinal bones, being generally classified into three groups: extradural, intradural-extramedullary, and intramedullary [25,26,27,28]. Among these categories, extradural tumors are the most frequently encountered, being commonly metastatic and occurring within vertebral bodies or structures outside the dura. The second most common spine tumors are the intradural-extramedullary ones, which come from leptomeninges or nerve roots. As their name implies, these tumors are located inside the dura but grow outside the spinal canal. The last category accounts for the least frequent type of spine tumors that arise from the spinal cord, which invade and destroy the gray and white matter [26,28].

More details are offered in the following subsections concerning spine tumors, dividing them according to their origin into primary spinal tumors and spinal metastases.

### 2.1. Primary Spinal Tumors

Primary spinal cord tumors represent a small proportion of all central nervous system tumors. Intramedullary spinal cord tumors (IMSCTs) make up a heterogeneous group of benign and malignant neoplastic tumors, including ependymoma, astrocytoma, glioblastoma, hemangioblastoma, ganglioglioma, germinoma, and lymphoma. Most IMSCTs do not locally infiltrate spinal cord parenchyma, and are classified as benign. Nonetheless, some tumors exhibit malignant behavior [29,30]. Unfortunately, they are often diagnosed in the late stages, especially when there are no neurological deficits in the early phases. When neurological symptoms occur, the tumor has already invaded the spinal canal, reducing the possibility of tumor resection and consequently causing significant morbidity and mortality [31].

Another category of spine-located tumors comprises primary osseous spinal tumors (POSTs), rare neoplasms accounting for about 5% of all primary bone tumors. The most frequent malignant POSTs are chordomas, chondrosarcomas, Ewing sarcomas, and osteosarcomas [32]. Chordomas represent the most frequent sacral malignancy, posing significant challenges from surgical and oncological perspectives. They are slow-growing bone-destructive tumors originating from primitive notochordal remnants of the axial skeleton that mostly occur in the sacrum, but can also arise in the mobile spine (e.g., cervical spine and thoracolumbar spine) [33,34,35]. In contrast, chondrosarcoma is most frequently encountered in the thoracic spine; however, it may also occur in other regions of the spine. Concerning their origin, chondrosarcomas primarily derive from the embryonic rest of the cartilaginous matrix [33,36]. Ewing sarcoma is one of the most prevalent bone sarcomas in young people, yet it rarely arises in the spine. However, when this mesenchymal tumor is present in the mobile spine, it poses major local treatment challenges, given its close proximity to neurologic and vascular structures [37,38,39,40]. The last-mentioned category of POSTs, spinal osteosarcomas, comprises tumors that are commonly located in the posterior elements of thoracic and sacral regions of the spine and, less frequently, in the cervical region. Most osteosarcoma symptoms are nonspecific, hindering diagnosis at an early stage of tumor development and consequently leading to a major burden on the patient, caregivers, and health-connected budgets [23].

### 2.2. Spinal Metastases

The spine represents the most common site of skeletal metastasis, often originating from prostate, lung, and breast cancers. Other less frequently reported primary malignancies that metastasize to the spine include colon, kidney, and upper gastrointestinal cancers [15,22,41,42]. Depending on their location, spinal metastases may be divided into three categories: intramedullary—located within the spinal cord; leptomeningeal—located within the subarachnoid space; and epidural metastases—located on the outside of the dura mater [15].

Spinal metastases affect a considerable number of cancer patients, causing debilitating symptoms and leading to significant morbidity in patients [15,22,41,42,43]. The growth of a spinal metastasis within the spinal canal may cause spinal cord compression, which may further lead to acute spinal cord injury and necessitate urgent surgical treatment. In addition, patients with spinal metastases may suffer from excruciating back pain, immobility, and neurological dysfunction, tremendously affecting their quality of life [42,43]. Thus, it is essential to initiate treatment as soon as possible to ensure proper recovery and functional rehabilitation.

## 3. Current Treatment Strategies and Their Limitations

Once a positive diagnosis has been made with the help of imaging techniques (e.g., plain radiography, computed tomography, myelography, magnetic resonance imaging, positron emission tomography) and histopathological examinations of a biopsy sample, a therapeutic strategy is proposed by a multidisciplinary tumor board [23,31]. However, when tumor management is urgent, multidisciplinary discussions risk creating treatment delays [44]; thus, decisions must be made quickly, especially for metastatic patients with acute neurological deficits. Furthermore, the management of complex spinal lesions is increasingly recognized to necessitate a concerted effort, encompassing the combined opportunities of radiotherapy, chemotherapy, and operative techniques [6,22]. Nonetheless, surgery has been demonstrated to be the most effective strategy in patients with neurological deficits and bone instability [22].

### 3.1. Surgical Treatment

The surgical excision of spine tumors represents the primary treatment option and must be integrated into the diagnostic and therapeutic strategy approved by the multidisciplinary tumor board [25,31,33,41]. The main indications for surgery include tumor control, decompression of the spinal cord, and mechanical stability restoration. The procedure goal differs with the type of tumor; namely, primary tumors are removed with a curative intent, while metastases are resected for symptom palliation [45]. However, tumor resection surgery is not feasible for multi-metastatic patients, and in such cases is usually replaced by decompression procedure [31].

A variety of surgical techniques can be employed for spine tumor resection, ranging from minimally invasive surgery to en bloc resection of affected tissues [15]. Surgical resection must combine tumor excision with a durable reconstruction of the spine and adjacent tissues [31]. In selected cases (i.e., vertebral compression fractures due to malignancy), percutaneous techniques, such as vertebroplasty and kyphoplasty, can be used for injecting polymethylmethacrylate into the vertebral body under X-ray or computed tomography. Polymer injection stabilizes vertebrae, reduces pain caused by microfractures, and prevents the vertebral body from further collapsing. However, these procedures are unsuitable for the pain or neurological deficits caused by nerve root or spinal compression because they do not contribute to tumor size reduction [15,46]. Using minimally invasive surgical techniques is a good alternative for facilitating postoperative recovery, decreasing the risk of complications, and quickening a patients’ return home and continuing oncological treatment. In this respect, endoscopic and robot-assisted procedures have gained ground for expanding tumor surgery capabilities [45].

However, tumor resection must be as complete as possible, given that it is nearly impossible to repeat excision procedures for primary malignant tumors of the thoracic and lumbar spine if the first resection is not complete [31]. In this context, surgical excision is often extensive in order to ensure the removal of all malignant tissue and prevent tumor recurrence [41]. Therefore, wide en bloc resection remains the most effective technique, implying the removal of the tumor surrounded by a layer of healthy tissue called the “margin”. Despite being vital for blocking tumor growth, the margin is often challenging to attain due to anatomic constraints. In such cases, postoperative adjuvant therapies are required to hinder tumor recurrence [23,33,34].

Resection of sacral lesions, in particular, is considered inherently difficult due to the structural role of the sacrum and the presence of the sacral nerve roots. Moreover, the close relationship between this anatomic structure and the pelvic vasculature poses the risk of intraoperative blood loss [34]. Generally, spine tumor resection consists of a double anterior plus posterior step and, less commonly, in a posterior approach alone. The anterior approach supposes sub-umbilical medial laparotomy to release the tumor anteriorly at the retrorectal space and enable peritumoral devascularization. On the other hand, the posterior approach may be executed in the same surgical step or 24 to 48 h after the anterior step, assuming a medial longitudinal or arched transverse incision that always includes the biopsy area [35].

Even though wide resection is crucial for ensuring local disease-free progression, it also requires more extensive reconstructive surgery and may lead to significant morbidity in the patient [15,34]. Surgical complications include surgical site infection, fatigue fracture (when surgery is combined with radiotherapy), hemorrhagic complications, pseudo-meningocele, osteomyelitis, sacroiliac instability, non-union, digestive fistula, cerebrospinal fluid leakage, and ureter wounds [35]. In particular, cerebrospinal leakage may further lead to a series of severe complications, counting infections (e.g., meningitis, arachnoiditis), intracranial hypotension-related issues (e.g., intracranial hemorrhage, cranial nerve palsies), and neurological deficits linked to the compression or incarceration of neural elements [47]. Moreover, anesthesiologists must be familiar with the associated perioperative risks and consider intraoperative neuromonitoring and patient comorbidities when establishing the anesthetic plan. Complications caused by intraoperative anesthetic factors and postoperative patient-controlled anesthesia may include delayed awakening, postoperative nausea, and vomiting. Other potential issues attributed to prolonged spinal tumor resection are postoperative visual loss (one of the rarest but most feared complications of spine surgery), acute or chronic pain, and pressure ulcers [27,48].

### 3.2. Non-Surgical Treatment

For some tumors, surgical treatment is either unsuitable or insufficient for efficiently managing spinal malignancies. Other approaches must be considered in such cases, with the most frequently encountered being chemotherapy, radiotherapy, and immunotherapy.

Chemotherapeutic drugs can be employed in treating advanced or unresectable tumors, in particular. The systemic delivery of paclitaxel, docetaxel, cisplatin, and doxorubicin represents a common practice for treating oligometastatic bone cancer [41]. Advances in developing chemotherapeutic protocols for spinal osteosarcoma have also been noted. Based mainly on the trials for osteosarcoma of the extremities, four drugs have been reported as effective: doxorubicin, cisplatin, high-dose methotrexate, and ifosfamide. In addition, recent research has recommended the use of a combined administration of antitumor agents to enhance their therapeutic potential and improve event-free survival rates [23].

However, despite their effectiveness in limiting tumor cell proliferation, chemotherapeutics must be administered in high systemic doses to reach tumor sites in adequate concentrations. This aspect negatively affects the health of normal tissues, leading to adverse effects such as neurotoxicity (paclitaxel), kidney toxicity (cisplatin), and cardiac toxicity (doxorubicin). A solution to this challenge would be to apply anticancer agents locally to deliver high doses on-site instead of subjecting the organism to systemic side effects [41]. Nonetheless, certain tumors, such as chondrosarcoma and chordomas, are not sensitive to chemotherapy. As no chemotherapy regimens have been standardized for these spinal malignancies, other therapeutic strategies must be considered [33]. Moreover, tumor cells may acquire resistance to chemotherapeutics, impeding their effects through various mechanisms (e.g., efflux, drug inactivation, alteration of drug targets, and cell death inhibition) [49]. The increased activity of efflux pumps in particular results in p-glycoprotein production, which is further responsible for transporting various anticancer drugs out of cells, leading to the appearance of multidrug resistance [50].

Radiotherapy represents a frequent treatment strategy, being implemented preoperatively, postoperatively, or exclusively when surgery is not possible [35]. Radiation therapy has been utilized for years, especially after subtotal tumor resection in intramedullary ependymomas or astrocytomas with conventional fractionation and doses of 45 to 50 Gy. The radiation dose is limited by the presence of the spinal cord and thoracic-abdominal organs so that the patient does not further suffer from radiation myelopathy and gastrointestinal or fertility issues [23,25]. In more detail, craniospinal irradiation was reported to affect the hormonal balance in women, negatively impacting their ability to become pregnant, while women treated with abdominopelvic radiation faced an increased rate of uterine dysfunction, resulting in miscarriage, preterm labor, low birth weight, and placental abnormalities [51]. Moreover, conventional radiotherapy may cause neurocognitive impairment in cancer survivors by damaging the neural progenitor populations responsible for adult hippocampal neurogenesis [52]. A recent alternative is stereotactic radiosurgery which has been rendered effective in patients with recurrent or residual disease, or in cases where surgery is contraindicated [25]. Other advanced radiotherapy modalities include proton beam therapy, carbon ion therapy, and intensity-modulated conformal radiotherapy, which are promising strategies for achieving precise localization and a sufficient radiation dose at the desired site in a shorter time. Thus, the exposure of nearby organs and surrounding healthy tissues to radiation is limited, while the pain and neurological symptoms are alleviated [23,34,35]. Nonetheless, several downsides still exist, including higher costs and potential sequelae (e.g., post-radiation pain, sensorimotor neuropathy, skin complications, and pathologic fractures) [15,35].

Another non-surgical treatment strategy that has increasingly been considered alongside chemotherapy and radiotherapy is immunotherapy [30,53]. Immunotherapeutic approaches have been oriented towards stimulating the patient’s immune system to selectively target and directly eliminate cancer cells instead of allowing them to evade or settle into an equilibrated status quo with the immune system [23,30]. Particular interest has been noted in applying immunotherapy to gliomas, with advances in the field including immune checkpoint inhibitors, chimeric antigen receptor (CAR) T therapy, and vaccine strategies. However, there are challenges hindering the application of immunotherapy to spinal cord gliomas, such as the low incidence, scarcity of targetable antigens, delivery across the blood–spinal cord barrier, immunosuppressive nature of the spinal cord tumor microenvironment, and neurotoxic treatment effects [30]. In addition, immune checkpoint inhibitors may produce negative regulation, causing autoimmune diseases and even death [54]. Vaccines have also been studied in relation to osteosarcoma, aiming to achieve antitumor effects through the exposure of tumor antigens of whole cells, lysates, proteins, DNA, RNA, or peptides. Specifically, dendritic cell vaccines were reported safe and feasible in relapsed osteosarcoma patients, yet only 2 out of 12 vaccinated patients exhibited a considerable antitumor response [23].

**Figure 1:**
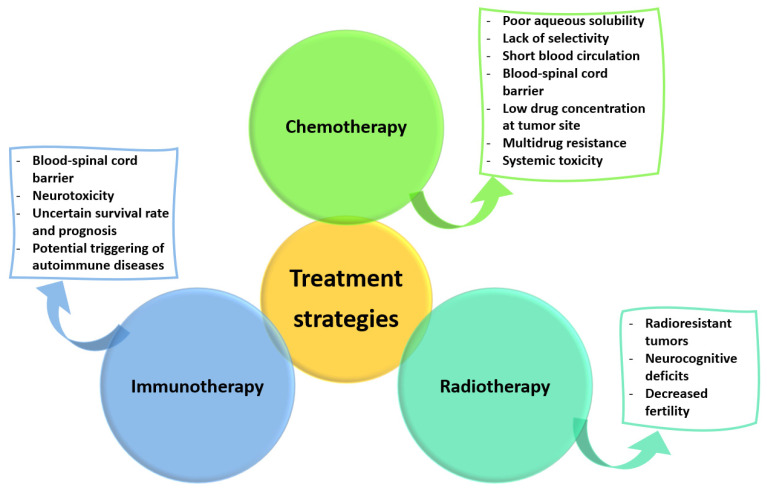
Limitations of current non-surgical treatment strategies. Created based on information from [30,49,50,51,52,54].

As all these therapeutic strategies present important limitations (Figure 1), better solutions must be envisaged for effectively managing spinal tumors.

## 4. Emerging Treatment Strategies

In a concerted effort to create more effective antitumor strategies, researchers worldwide proposed interesting alternatives for improving the available therapeutic approaches, reconstruction strategies, and surgical procedures involved in spine tumor management. In this respect, the following subsections review the novelties in these fields, as well as presenting the advances in spine tumor treatment that have reached clinical testing.

### 4.1. Tumor-Targeted Therapies

Investigation of the local environment of neoplastic diseases in recent decades has led to the discovery of the tumor microenvironment (TME) and its characteristics, which further allowed a more in-depth understanding of cancer progression and the subsequential development of more specific therapies.

TME can be regarded as the “soil” for cancer development, encompassing irregular vasculature, dense stroma, and unique cellular and noncellular components. In more detail, TME involves neoplastic cells (e.g., cancer stem cells, cancer-associated fibroblasts), infiltrating cells (e.g., lymphocytes, tumor-associated macrophages), and resident cells (e.g., fibroblasts, endothelial cells) that remodel the extracellular matrix (ECM). Tumor–tumor cell communication, tumor–stromal cell communication, and tumor–ECM interface have all been noted to contribute to direct cell interaction mediated by drug resistance. In addition, the complex cocktail of growth factors and cytokines produced in the TME controls the progression of cancer and results in local hypoxia, hypoglycemia, and acidosis [29,55,56,57].

Even though the role of TME in cancer progression is well described for many malignancies, the knowledge of central nervous system TME is still incomplete, leading to a poor understanding of IMSCTs pathology. Moreover, the rarity of these tumors resulted in neuro-oncology being almost exclusively focused on cerebral tumor biology, neglecting spinal cord tumor biology and leaving a research gap that must be filled to allow for the development of targeted therapeutic approaches [29].

As tumor cells overexpress specific cell surface receptors, they can be exploited as targets for antibodies or smaller molecules to enable cytotoxic compound delivery to tumor cells [58]. TME characteristics can also be leveraged in the efficient management of cancer by providing responsiveness opportunities to nanobiotechnological modalities to release anticancer agents under various stimuli, such as enzymes, temperature, pH, redox potential, or other external stimuli based on their distinct physicochemical parameters [55].

In contrast to the tumor microenvironment of intrinsic bone/spinal cord tumors, the metastatic niche can be targeted instead for spinally metastasizing tumors. The metastatic bone niche assumes a unique combination of cell types, connective tissues, signaling molecules, trophic factors, cytokines, and chemokines. The metastatic niche is mainly represented by the presence of tumor-derived growth factors in response to which tumor-associated immune cells cluster at the distant metastatic site, preparing the “soil” for the arrival of cancer cells and facilitating their adhesion and proliferation. A deep understanding of the metastatic niche is key to creating targeted treatment strategies for spinal metastasis, with few examples of effective therapeutic agents already known (i.e., osteoclast-targeting bisphosphonates and the RANKL-neutralizing antibody) [59,60].

**Figure 2:**
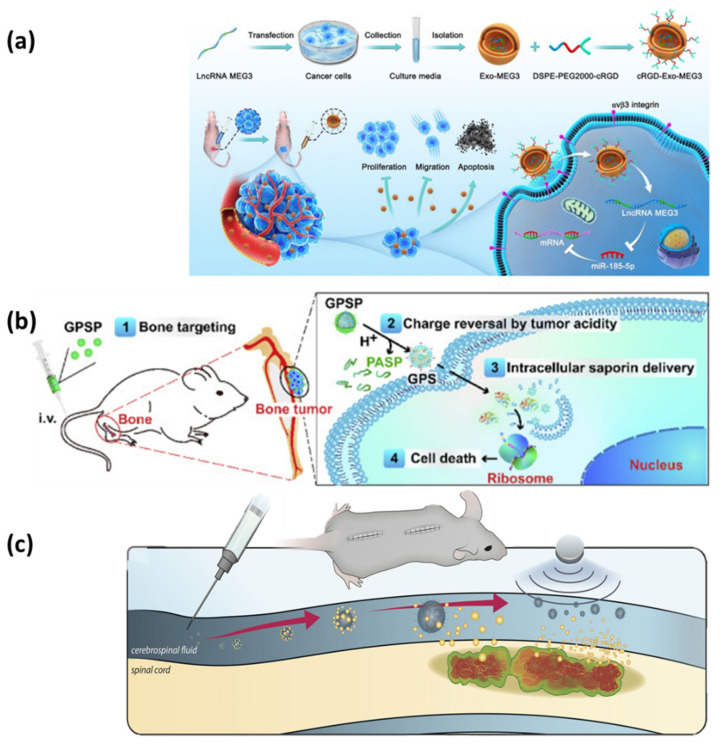
(**a**) Schematic representation of fabrication and working principle of the experimental design employed by Huang et al. Reprinted with permission from [66], © Elsevier, 2022. (**b**) Schematic representation of the working principle of the experimental design employed by Yan et al. Adapted from an open-access source [61]. (**c**) Schematic representation of the working principle of the experimental design employed by Kheirkhah et al. Adapted from an open-access source [70].

In this context, several targeted-delivery nanoformulations have been recently developed as promising tools against POSTs and spinal metastases. For example, Yan et al. [61] have recently conducted a study on mice to investigate their novel tumor-targeted delivery formulation. The scientists have prepared a bone-targeted protein nanomedicine consisting of saporin co-assembled with a boronated polymer and coated with an anionic poly(aspartic acid) layer. The nanoparticles successfully accumulated in the bone and, triggered by tumor extracellular acidity, released saporin into the tumor cells, inactivating ribosomes and leading to cancer-cell death (Figure 2b). Thus, the developed nanomedicine could destroy tumor cells at a low chemotherapeutic dose, preventing the progression of osteosarcoma xenograft tumors and bone metastatic breast cancer.

In another study, Wu et al. [62] have fabricated alendronate and low molecular weight heparin-modified liposomes for the delivery of doxorubicin. The authors chose alendronate as a bone-targeting moiety while low molecular weight heparin was included to enhance the blood circulation time of liposomes and exhibit anti-metastasis efficiency. The proposed system was able to suppress tumor growth and inhibit tumor metastasis in mice, and is a promising therapeutic approach against both osteosarcoma and bone metastases.

Ahmadi et al. [63] have alternatively studied their innovative anticancer formulation on the Saos-2 human osteosarcoma cell line. The researchers reported the delivery of methotrexate encapsulated into a smart nanocarrier made of a magnetic inner core and polymeric outer shell with cationic moieties. The magnetic delivery system was also observed to exhibit a pH-responsive release, successfully internalizing into tumor cells and demonstrating higher cytotoxicity than the free drug.

The same cell line was targeted by Khelghati and colleagues [64], who have developed a pH-sensitive magnetic hyperbranched β-cyclodextrin as a nanocarrier for doxorubicin. The as-designed nanosystem proved higher cytotoxicity than doxorubicin alone, and is a promising biocompatible solution for doxorubicin delivery to the Saos-2 cell line.

Alternatively, Plesselova et al. [65] have created a polyethyleneimine scaffold conjugated with bisphosphonates as targeting ligands and cyclodextrins as supramolecular drug carriers. The authors tested this nanovehicle for the delivery of doxorubicin to three different bone-related cancer cell lines (i.e., MC3T3-E1 osteoblasts, MG-63 sarcoma cells, and MDA-MB-231 breast cancer cells). Investigations demonstrated desirable properties for the system, counting specificity, mitochondrial targeting, and the ability to ensure drug transport to tumor cells.

On a different note, Huang et al. [66] have constructed nanoparticles that combine the benefits of exosomes and lncRNA MEG3 for the tumor targeting of four human osteosarcoma cell lines (i.e., MNNG/HOS, U2OS, MG63, and SaOS-2). The engineered nanosystems could deliver more efficiently to osteosarcoma cells, facilitating the antitumor effects of MEG3, and enhancing its therapeutic potential (Figure 2a).

Xiao et al. [67] have also prepared an RNA nanoparticle delivery system, but this research group focused on targeting human chordoma cell line U-CH2 instead. For this purpose, the authors engineered paclitaxel-loaded 3-way junction nanoparticles harboring the specific EGFR-targeting RNA aptamer and the Alexa Fluor-647 imaging modules. The system demonstrated excellent binding and localization to EGFR(+) U-CH2 cells in vitro, whereas it failed to bind EGFR(−) H520. Therefore, the formulation improves the drug’s solubility, ensures a targeted drug delivery to the desired cells, and enhances tumor cell inhibition.

Another paclitaxel delivery system was designed by Yang et al. [68], who used a bone metastasis-targeted glutamic hexapeptide-folic acid (Glu6-FA) derivative as a ligand attached to liposomes. The Glu6-FA modified liposomes exhibited superior targeting ability in vitro and in vivo compared to free paclitaxel, non-coated, and single-modified liposomes, and are excellent vehicles for drug delivery to metastatic bone cancer.

Pham et al. have also [69] created a nanosystem for targeting bone metastatic breast cancer. For this purpose, the authors delivered doxorubicin via alendronate-functionalized graphene oxide nanosheets, achieving longer retention and higher concentrations in bone tumor areas than for free drug and non-functionalized material. Thus, it was concluded that these nanosystems represent viable candidates to augment the antitumor effects and reduce the off-target toxicities of chemotherapeutic drugs.

An innovative treatment strategy has also been developed for IMSCTs. Specifically, Kheirkhah et al. [70] have synthesized magnetic nanoparticles loaded with doxorubicin for the concentrated delivery to intramedullary spinal cord tumor models. Using magnetism as a physical stimulus, the authors managed to direct the delivery system to the desired site, avoiding the toxicity associated with systemic drug administration (Figure 2c). The nanoparticles demonstrated focal, chemotherapeutic-induced apoptosis of cancer cells, and are promising candidates for antitumor treatment.

To summarize the above-discussed studies, Table 1 correlates the nanocarrier, transported chemotherapeutic, mechanisms of action, and targeted tumor type.

### 4.2. Custom-Made Vertebral Body Implants

Surgical spine oncology often supposes unique spatial reconstruction that aims to restore spinal length, alignment, and weight-bearing capacity while providing immediate stability to the spine [17,71]. In the last few years, 3D printing technologies have gained tremendous popularity, starting to be considered in spinal oncology for the manufacture of custom-made vertebral body implants. The engagement of these additive manufacturing techniques begins with virtually constructing the design with the aid of designated software to match the specific shape required by the patient, as identified from computed tomography or magnetic resonance imaging [24,72,73]. The utilization of personalized implants brings a series of advantages, including reduced operative times, reduced blood loss, immediate stability, improved fusion rates, lower rates of pseudoarthrosis, ensuring spinal homeostasis, and increased procedure success rates due to proper osseointegration [71,72,74]. Taking into account these benefits, researchers have incorporated 3D-printed customizable scaffolds into the treatment plan for a series of spine tumors, obtaining promising results.

For instance, Xu and colleagues [75] have utilized a customized implant in the case of a young boy with Ewing sarcoma who underwent a staged spondylectomy. The artificial vertebral body was manufactured according to a computer model, and its microstructure was optimized to ensure better mechanical stability and enhanced bone healing. The procedure was successful, the osseointegration of the implant occurred, no subsidence or displacement was observed, and the patient was tumor-free at the 1-year follow-up.

Another case of the application of a custom-made vertebral implant in the cervical spine has been described by Parr et al. [76]. The researchers used this reconstruction strategy following the resection of a C3–C5 chordoma tumor to optimize patient outcomes through maximized anatomic integration. The implant design included particularized features, such as a sagittal plane anatomic curvature, zero anterior profile relief, patient-matched anatomic endplate contact surfaces, smooth transition from a smaller to larger device footprint, and preplanned trajectories for fixation screws, thus improving both surgical implantation and postoperative functionality.

Wei and colleagues [77] have also employed 3D-printed vertebral implants for upper cervical reconstruction. The authors have tackled this approach in nine patients with primary tumors involving C2, concluding that it is a reliable method for spinal reconstruction. In more detail, the personalized shape matching with the contact surfaces and the porous structure conductive to osseointegration managed to offer stability to the implant in both the short and long terms.

Alternatively, Wang et al. [78] have presented the reconstruction of the thoracolumbar spine following a one-stage en bloc spondylectomy of multi-segment thoracolumbar metastasis. In this respect, they have developed customized columnar 3D-printed artificial vertebrae that match the patient’s physiological curvature and the contact surfaces of the upper and lower vertebrae end plates. The proposed design is promising for increasing implant stability while decreasing the risks of sagittal spinal misalignment, displacement, and subsidence.

Mobbs et al. [74] have reported the use of a patient-specific implant in the lumbar spine. The implant allowed for a superior anatomical fit and significantly reduced the operative time. These aspects would be reflected in the longer term by reduced subsidence, reduced radiation exposure, and reduced infection risk.

A different case is presented by Chatain and Finn [72], who have used a personalized 3D-printed sacral implant for the reconstruction of the spinopelvic continuity after sacral resection. The patient underwent a total en bloc sacrectomy due to sacral chordoma, and the standard reconstruction procedure failed. Thus, scientists proposed the implantation of a custom-made sacral prosthesis as a salvage reconstruction surgery. The artificial sacrum was believed to be critical for the patient, ensuring enough biomechanical stability to promote healing and allowing bony incorporation into the graft. However, the procedure was considered difficult from the surgeons’ point of view.

Kim et al. [79] have reported the use of a 3D-printed implant in the case of sacral osteosarcoma surgically treated with hemisacrectomy. This strategy has led to significantly reduced postoperative pain and allowed a fast recovery, with the patient being able to walk 2 weeks after the procedure. Given the excellent bony fusion observed 1 year after implantation, the authors concluded that this strategy is promising for spinal reconstruction and suitable for various spinal diseases.

Promising results can also be obtained when using such customized scaffolds as drug delivery systems [80]. In this regard, Ahangar et al. [41] have designed a series of 3D-printed structures loaded with doxorubicin for the local treatment of spine metastases. The researchers incorporated the chemotherapeutic drug into highly porous thermoplastic polyurethane scaffolds, creating low-cost platforms that could fill bone defects after tumor resection while also limiting cancer recurrence.

To offer an at-a-glance perspective on the above-presented studies, Table 2 synthesizes the discussed information, including details on the material and site of the vertebral body implants and some postoperative observations.

Overall, custom-made vertebral body implants are considered a promising reconstruction strategy, offering better outcomes for treated patients in terms of mobility, recovery time, and pain relief. The reduced implantation time that is achievable with these devices also contributes to the reduction of open wound duration, diminishing the infection risk [74]. Nonetheless, despite their increasing popularity, larger series of customized implants should be studied and compared to commercially available alternatives in order to determine their added value [17].

Even though fabricating a personalized 3D-printed implant is more expensive than a generic device, customized artificial vertebral bodies bring cost-effectiveness to the overall procedure. In more detail, such custom-made implants can more rapidly be introduced at the desired site, reducing the time spent in the operating theater and the associated costs. Moreover, as the production workflow of these devices develops and matures, manufacturing costs are expected to decrease [74].

### 4.3. Surgical Novelties

The outcomes of spine tumor treatments can also be improved by enhancing surgical procedures with the aid of recent technological advances. Many forms of navigation have already been involved in spine surgery, including guiding freehand techniques by fluoroscopy, 3-dimensional navigation, and stereotaxis. However, more recently, scientific interest gathered around the use of emerging augmented reality (AR), virtual reality (VR), and mixed reality (MR) technologies [16,81,82,83].

Despite having similar purposes, these technologies must be distinguished from the point of view of their functionality. AR utilizes computer and imaging technology to overlap with transparent, patient-specific anatomy and pathology directly onto the surgical field, while VR completely replaces the operating environment with a computer-generated one, immersing the surgeon in a virtual representation of the real surgical field. Alternatively, MR proposes any combination of interacting real and virtual environments [16]. Numerous recent studies [84,85,86,87,88,89,90,91] have reported favorable results with the help of AR, VR, and/or MR compared with conventional approaches when treating patients with various spinal pathologies, including tumor resection. Moreover, advanced navigation technologies can be employed in constructing a 3-dimensional representation of the spine, thus providing real-time positional feedback during the operation and visualization of deeper structures [92].

Surgical procedures can also be improved through the utilization of artificial intelligence (AI)-powered image guidance for directing constructs and avoiding iatrogenic injury. For instance, computer-assisted navigation (CAN) platforms have already found application in many operating rooms in the United States, being involved in different procedures, including the resection of spinal tumors. CAN is considered a promising approach for improving the accuracy of operative tasks and efficiency of operations, reducing the duration of generalized anesthesia, and decreasing the risks of complications [92,93].

In addition, AI tools such as deep neural networks can be applied to predict surgery outcomes and postoperative complications. In particular, research was directed towards employing certain models that, based on a number of variables, could accurately predict spine infections [94,95].

### 4.4. Clinical Trials

Several new approaches to dealing with spinal tumors have reached the stage of clinical testing. In this respect, Table 3 summarizes the clinical studies that have been either completed or terminated and have results posted on ClinicalTrials.gov.

From the above-tabulated studies, a few of them have been interpreted in more detail in journal publications. For instance, Redmond et al. [107] extensively discussed the results of clinical trial NCT01752036 concerning the use of postoperative SBRT for solid tumor spine metastases. The tested treatment strategy demonstrated excellent local control with low toxicity, and is associated with superior rates of local control to conventional radiotherapy. Nonetheless, a formal comparative study should be performed for confirmation.

The results of NCT00922974 have been presented by Ryu et al. [108]. The study has shown the feasibility, accuracy, and early safety of performing single-fraction image-guided stereotactic radiosurgery to treat spinal metastases, exhibiting rigorous quality assurance in a cooperative group setting. The authors concluded that the investigated method has the potential to become a standard of care in managing localized spine metastases with or without spinal cord compression.

More recently, Levy and colleagues [109] described the results of clinical trial NCT03249584. All the ablations performed in this study were technically successful, with 97% being followed by cementoplasty. Patients reported improvements in average pain, pain interference, and quality of life. Even though a number of subjects died, their death was not related to the procedure but to the underlying malignancy. Thus, it was considered that radiofrequency ablation represents a viable treatment strategy for axial skeletal metastases, ensuring rapid and long-lasting significant pain relief.

Moreover, interesting prospects might soon come from undergoing clinical trials. In this regard, a summary of active studies has been documented in Table 4, emphasizing the increasing research interest in this field.

## 5. Conclusions and Future Perspectives

To conclude, whether they are primary tumors or metastases of advanced cancers originating elsewhere in the body, spine tumors represent serious conditions requiring prompt multidisciplinary treatment approaches. Hence, recent research interest has been noted in improving conventional treatment strategies by the targeted delivery of drugs via engineered nanosystems, designing custom-made vertebral body implants to repair bone defects resulting from tumor resection, as well as improving surgical precision, reducing operative time, and lowering the risk of postoperative complications through the use of AR, VR, and MR technologies. Interesting possibilities may arise from combined approaches using targeted therapies, personalized implants, and surgical novelties, yet no study has been found to investigate the three-fold perspective at the moment.

Given the rarity of primary spine tumors compared to other malignancies, these diseases are often neglected in research. However, their complexity and severe sequelae ask for a more in-depth investigation of tumor mechanisms within the spinal cord. Specifically, the knowledge of spinal cord tumor biology must be extended to better understand the involvement of infiltrating stromal cells in the pathology of IMSCTs [29] and further allow TME-tailored therapeutic approaches. Further research must also be considered for assessing the long-term outcomes of discussed treatment strategies, optimizing recently developed formulations, advancing from animal models to human studies, and implementing already clinically tested interventions into practice.

Moreover, new technologies such as artificial intelligence [131] and big data analytics [132] hold promise for improving the quality of care for patients with spine tumors. These novel instruments can help assess the molecular markers of spine tumors, predict the survival of primary spine tumors or metastatic recurrence rates, and guide clinical decision-making.

In closing, given the encouraging results of recent studies and clinical trials, it can be expected that the interdisciplinary approach of medicine, material science, nanotechnology, and computer science would lead to the development of successful treatment strategies against spine tumors.

## Figures and Tables

**Table 1:** Summary of discussed tumor-targeting therapies.

Nanocarrier	Chemotherapeutic	Mechanism(s) of Action	Tumor Type	Reference
Boronated polymer coated with anionics poly(aspartic acid)	Saporin	- the anionic layer provides bone targeting function - release triggered by tumor extracellular acidity - the boronated polymer promotes intracellular delivery - saporin inactivates ribosomes	Osteosarcoma	[61]
Liposomes modified with alendronate and low molecular weight heparin	Doxorubicin	- alendronate provides bone-targeting function - heparin enhances blood circulation time - heparin also inhibits tumor metastasis-related enzymes, cell adhesion, and tumor neovascularization	Osteosarcoma Breast cancer bone metastasis	[62]
Magnetic nanoparticles coated with cationic cyclodextrin	Methotrexate	- pH-responsive drug release - explosive and fast release of cargo prevents developing resistance - non-endosomal cell entry helps avoid endo/lysosome entrapment of internalized particles and transport drugs efficiently into the cytoplasm	Osteosarcoma	[63]
Magnetic hyperbranched β-cyclodextrin	Doxorubicin	- pH-responsive drug release - following cell internalization, the system causes shrinkage, fragmentation, and perforation of nucleus	Osteosarcoma	[64]
Polyethyleneimine–bisphosphonate (BP)–cyclodextrin ternary conjugates	Doxorubicin	- BP conjugation increases system’s affinity to hydroxyapatite - the nanosystem significantly blocks ERK1/2 signaling pathways that promote tumor cell proliferation	Osteosarcoma Breast cancer bone metastasis	[65]
c(RGDyK)-modified exosomes	lncRNA MEG3	- cRGD modification enhanced tumor-targeting ability and accumulation - upregulation of MEG3 inhibits cell proliferation and migration and promotes tumor cell apoptosis	Osteosarcoma	[66]
3-way junction nanoparticle functionalized with EGFR aptamer	Paclitaxel	- the aptamer provides tumor-targeting ability, excellent binding and internalization into selected cells	Chordoma	[67]
Liposome modified with glutamic hexapeptide-folic acid	Paclitaxel	- functionalization agents provide hydroxyapatite affinity, ensuring bone-targeting ability - the nanosystem produces cell cycle arrest, blocking tumor cell proliferation	Breast cancer bone metastasis	[68]
Alendronate-functionalized graphene oxide nanosheets	Doxorubicin	- alendronate provides bone-targeting function - pH-responsive drug release - interaction with the skeletal system and confinement of nanosheets to bone contribute to prolonging in vivo retention, delaying entrapment by reticuloendothelial system and accessibility to the glomerular filtration	Breast cancer bone metastasis	[69]
Magnetic nanoparticles	Doxorubicin	- magnetic drug targeting (nanosystems are manipulated under the influence of an external magnetic field) - pH-responsive drug release	High-grade intramedullary spinal cord tumors	[70]

**Table 2:** Summary of discussed custom-made vertebral body implants.

Implant Material	Implant Site	Tumor Type	Postoperative Observations	Reference
Titanium alloy	Upper cervical spine (between C1 and C3)	Ewing sarcoma	Uneventful recovery The patient began to ambulate on postoperative day 7 and started adjuvant treatment 3 weeks after surgery Tumor-free at the 1-year follow-up	[75]
Titanium alloy	Cervical spine (between C2 and C6)	Chordoma	No postoperative complications The patient was mobilized 48 h after the anterior intervention, was discharged after 10 days, and was required to wear a neck brace for 10 days No change in implant position, no evidence of hardware failure, and no significant adverse effects at 14 months follow-up Tumor-free at the 15 months follow-up	[76]
Titanium alloy	Upper cervical spine	Primary osseous spinal tumors	No sign of displacement or subsidence During a mean follow-up of 28.6 months, 1 patient died of systemic metastasis, and 1 had local tumor recurrence, while the other 7 patients were alive and functional in their daily living	[77]
Titanium alloy	Thoracolumbar spine (between T10 and L2)	Breast cancer metastasis	The patient was stable 3 days after the operation, and after 6 days, the back pain was significantly alleviated; the patient could walk normally independently with a thoracolumbar brace Tumor-free at the 2 years follow-up	[78]
Titanium alloy	Lumbar spine (between L4 and S1)	Renal cell metastasis	No intraoperative complications The patient was mobilized on postoperative day 4 and discharged on day 15 At the 3-month follow-up, the surgical and low back pain settled considerably, but the functional motion range of the lumbar spine remained limited	[74]
Porous titanium mesh	Sacrum	Chordoma	Tumor-free at the 18-month follow-up The patient could walk short distances with assistance	[72]
Porous titanium mesh	Sacrum	Osteosarcoma	The patient could walk 2 weeks after surgery Due to the resection of the left S1 nerve root, there occurred a left foot drop and neuropathic pain in the left leg The patient underwent 3 cycles of adjuvant chemotherapy up to 12 months after the surgery	[79]

**Table 3:** Summary of completed clinical studies concerning spine tumors. Information retrieved from ClinicalTrials.gov using the following search constraints: “Spine tumor”—with results.

ClinicalTrials.gov Identifier	Official Title	Intervention/Treatment	Phase	Actual Study Completion Date	Reference
NCT00593320	Stereotactic Radiosurgery (SRS) for One or Two Localized Spine Metastases	Radiation: Stereotactic Radiosurgery	Not Applicable	March 2010	[96]
NCT01525745	Randomized Ph II Study of Stereotactic Body Radiotherapy (SBRT) Versus Conventional Radiation for Spine Metastasis	Radiation: Radiosurgery/SBRT Radiation: External Beam Radiation Therapy	Phase 2	January 2014	[97]
NCT03050203	Surgical Field Custom Pack’s Efficacy on Soft Tissue Dissecting Time Reduction, on Relative Risks and Materials Wasted, in Patients Undergoing Spine Surgery: Randomized Controlled Trial	Other: custom pack Other: standard care	Not Applicable	30 July 2016	[98]
NCT01654068	Conformal High Dose Intensity Modulated Radiation Therapy for Asymptomatic Metastatic Disease to the Thoracic and Lumbar Spine	Radiation: Conformal High Dose Intensity Modulated Radiation Therapy	Not Applicable	8 September 2016	[99]
NCT01757717	A Pilot Study of Image-Guided Navigation for High Dose Rate Temporary Interstitial Brachytherapy in the Palliative Management of Previously Treated Tumors of the Spine and Pelvis	Radiation: Ir-192 high dose rate (HDR)	Not Applicable	July 2017	[100]
NCT01347307	Phase IV Trial Evaluating the Use of Stereotactic Body Radiotherapy for the Treatment of Spine Metastases and Primary Spine Tumors	Radiation: SBRT for Benign Extradural Spine Tumors Radiation: SBRT for Vertebral/Paraspinal Metastases	Not Applicable	September 2017	[101]
NCT01752036	Phase II Study of Postoperative Stereotactic Radiosurgery for Solid Tumor Spine Metastases	Radiation: Postoperative, SBRT	Phase 2	27 July 2018	[102]
NCT00922974	Phase II/III Study of Image-Guided Radiosurgery/SBRT for Localized Spine Metastasis	Radiation: External beam radiation therapy Radiation: Radiosurgery/SBRT	Phase 2 Phase 3	6 April 2020	[103]
NCT03249584	OsteoCool Tumor Ablation Post-Market Study (OPuS One)	Device: OsteoCool™ RF Ablation	Not Applicable	17 July 2020	[104]
NCT00855803	Phase II Study of Stereotactic Body Radiation Therapy and Vertebroplasty for Localized Spinal Metastasis (SBRT Spine)	Radiation: radiation	Phase 2	20 January 2021	[105]
NCT02085941	Image-guided Cryoablation of Head, Neck and Spine Tumors	Device: Cryoablation Device: Biopsy	Not Applicable	July 2021	[106]

Abbreviations: SBRT—stereotactic body radiotherapy; RF—radiofrequency.

**Table 4:** Summary of active clinical studies concerning spine tumors. Information retrieved from ClinicalTrials.gov using the following search constraints: “Spine tumor”–Recruiting; Not yet recruiting; Active, not recruiting; Interventional.

ClinicalTrials.gov Identifier	Official Title	Intervention/Treatment	Phase	Estimated Study Completion Date	Reference
NCT04578691	A Two-arm, Single Center, Randomised Study to Evaluate the Safety and Clinical Outcome of Using Navigation System in Pedicle Screw Placement in Spine Surgery	Device: “Anatase” Spine Surgery Navigation System Device: Medtronic Stealthstation S7 Treatment Guidance System	Not Applicable	31 December 2021	[110]
NCT00508443	Phase I/II Evaluation of a Novel CT-On-Rails or Trilogy Stereotactic Spine Radiotherapy System (SSRS) for the Treatment of Metastatic Spine Disease	Radiation: Radiation Therapy	Phase 1 Phase 2	31 October 2022	[111]
NCT02987153	Kypho-Intra Operative Radiation Therapy (IORT) for Localized Spine Metastasis, Phase I/II Study	Radiation: Kypho-IORT	Not Applicable	November 2022	[112]
NCT02387905	Prophylactic Cement Augmentation for Patients at High Risk for Developing Vertebral Body Compression Fracture Following Spine Stereotactic Radiosurgery: A Randomized Phase II Clinical Trial	Procedure: Management of Therapy Complications Other: Quality-of-Life Assessment Other: Questionnaire Administration Radiation: Stereotactic Radiosurgery	Phase 2	30 November 2022	[113]
NCT05174026	A Pilot Study on the Efficacy of Advanced 18F-FDG PET-MRI in Spine Stereotactic Radiosurgery	Other: Fludeoxyglucose F-18 Procedure: Magnetic Resonance Imaging Procedure: Positron Emission Tomography	Not Applicable	31 December 2022	[114]
NCT03575949	Dual-Time Point (DTP) FDG PET CT for the Post-Treatment Assessment of Head and Neck Tumors Following Definitive Chemoradiation Therapy	Procedure: Computed Tomography Other: Fludeoxyglucose F-18 Procedure: Positron Emission Tomography	Not Applicable	31 December 2022	[115]
NCT03028337	Single Versus Multifraction Salvage Spine Stereotactic Radiosurgery for Previously Irradiated Spinal Metastases: A Randomized Phase II Clinical Trial	Radiation: Spine Radiosurgery Behavioral: Questionnaires	Phase 2	18 January 2023	[116]
NCT04635137	Percutaneous Ablation and Cementoplasty for Painful Bone Lesions: A Canadian Single-Centre Experience	Procedure: Ablation and Cementoplasty	Not Applicable	March 2023	[117]
NCT05204290	A Pilot Study of Combined Decompressive Spine Radiosurgery and Pembrolizumab in Patients with High-Grade Epidural Disease	Drug: Pembrolizumab Radiation: Stereotactic Body Radiation Therapy Other: Blood draws	Early Phase 1	September 2023	[118]
NCT05280067	Feasibility Study of ZetaFuse™ Bone Graft in the Repair of Bone Defects from Metastatic Breast Cancer in the Spinal Vertebral Body	Device: ZetaFuse™ Bone Graft	Not Applicable	September 2023	[119]
NCT05493228	The Role of Dexmedetomidine (Precedex) Infusion on Intraoperative Propofol & Fentanyl Requirements in Spine Surgery for Pediatric Cancer Patients	Drug: Precedex Injectable Product Drug: Saline	Phase 3	30 October 2023	[120]
NCT05467540	Clinical Study of SPINERY™ A Novel Radio-Frequency Tumor Ablation Device for Spine Metastatic Tumors	Device: SPINERY	Not Applicable	30 November 2023	[121]
NCT04218617	Single- vs. Two-Fraction Spine Stereotactic Radiosurgery for the Treatment of Vertebral Metastases	Device: Diagnostic MRI Device: Planning MRI Other: Simulation CT Other: QOL assessment Other: Brief pain inventory (BPI) Radiation: sSRS in 1 fraction Radiation: sSRS in 2 fraction	Phase 2	1 January 2024	[122]
NCT05427825	Anesthetic Protocols for Enhance Recovery After Metastatic Spine Tumor Resection Surgery: A Randomized Controlled Trial	Other: ERAS anesthetic care Other: Standard anesthetic care	Not Applicable	January 2024	[123]
NCT04033536	A Prospective Randomized Trial of Involved Versus Elective Target Definition in Stereotactic Spine Radiosurgery for Spinal Metastases	Radiation: Involved Target Stereotactic Spine Radiosurgery Radiation: Elective Target Stereotactic Spine Radiosurgery	Not Applicable	June 2024	[124]
NCT04375891	Randomized Phase II Study of Radiation Therapy Alone Versus Radiation Therapy Plus Radiofrequency Ablation (RFA)/Vertebral Augmentation for Localized Spine Metastasis	Radiation: Radiation Therapy Radiation: Radiofrequency Ablation (RFA)	Not Applicable	1 September 2024	[125]
NCT05023772	A Clinical Trial Evaluating the Efficacy of Combining Laser Interstitial Thermal Ablation with and Without Spine Stereotactic Radiosurgery for Patients with Spine Metastases	Procedure: Stereotactic Laser Ablation Radiation: Stereotactic Radiosurgery Diagnostic Test: MRI guided laser ablation	Not Applicable	September 2024	[126]
NCT05396222	A Prospective Study of the Safety and Efficacy of 3D-printed Custom-made Non-rigid Biomimetic Implant for Anterior Column Reconstruction in Cervical and Thoracolumbar Spine	Device: 3D-printed custom-made non-rigid biomimetic implant	Not Applicable	4 February 2025	[127]
NCT05317026	Pre-irradiation Vertebroplasty in Patients with Spine Metastases Candidates for SBRT vs. SBRT Alone: Increased Early Pain Relief	Procedure: Vertebroplasty Procedure: Stereotactic Body Radiation Therapy only	Not Applicable	31 December 2025	[128]
NCT04802603	Dose-Escalated Spine SbRT (DESSRT) for Localized Metastasis to the Spinal Column	Radiation: Spine stereotactic body radiotherapy	Not Applicable	31 December 2026	[129]
NCT05495399	Surgery for Limited Spine Metastases Followed by Conventional Radiotherapy or Stereotactic Body Radiation Therapy	Procedure: Spondylectomy Radiation: SBRT	Not Applicable	July 2027	[130]

## Data Availability

Not applicable.
